# Differential Iron Requirements for Osteoblast and Adipocyte Differentiation

**DOI:** 10.1002/jbm4.10529

**Published:** 2021-07-26

**Authors:** Daniel F. Edwards, Christopher J. Miller, Arelis Quintana‐Martinez, Christian S. Wright, Matthew Prideaux, Gerald J. Atkins, William R. Thompson, Erica L. Clinkenbeard

**Affiliations:** ^1^ Department of Medical and Molecular Genetics School of Medicine, Indiana University Indianapolis IN USA; ^2^ Department of Physical Therapy School of Health & Human Sciences, Indiana University Indianapolis IN USA; ^3^ Indiana Center for Musculoskeletal Health Indiana University Indianapolis IN USA; ^4^ Centre for Orthopaedic & Trauma Research University of Adelaide Adelaide South Australia Australia

**Keywords:** ADIPOCYTE, IRON, MESENCHYMAL STROMAL CELLS, OSTEOBLAST

## Abstract

Bone marrow mesenchymal progenitor cells are precursors for various cell types including osteoblasts, adipocytes, and chondrocytes. The external environment and signals act to direct the pathway of differentiation. Importantly, situations such as aging and chronic kidney disease display alterations in the balance of osteoblast and adipocyte differentiation, adversely affecting bone integrity. Iron deficiency, which can often occur during aging and chronic kidney disease, is associated with reduced bone density. The purpose of this study was to assess the effects of iron deficiency on the capacity of progenitor cell differentiation pathways. Mouse and human progenitor cells, differentiated under standard osteoblast and adipocyte protocols in the presence of the iron chelator deferoxamine (DFO), were used. Under osteogenic conditions, 5μM DFO significantly impaired expression of critical osteoblast genes, including osteocalcin, type 1 collagen, and dentin matrix protein 1. This led to a reduction in alkaline phosphatase activity and impaired mineralization. Despite prolonged exposure to chronic iron deficiency, cells retained viability as well as normal hypoxic responses with significant increases in transferrin receptor and protein accumulation of hypoxia inducible factor 1α. Similar concentrations of DFO were used when cells were maintained in adipogenic conditions. In contrast to osteoblast differentiation, DFO modestly suppressed adipocyte gene expression of peroxisome‐proliferating activated receptor gamma, lipoprotein lipase, and adiponectin at earlier time points with normalization at later stages. Lipid accumulation was also similar in all conditions. These data suggest the critical importance of iron in osteoblast differentiation, and as long as the external stimuli are present, iron deficiency does not impede adipogenesis. © 2021 The Authors. *JBMR Plus* published by Wiley Periodicals LLC on behalf of American Society for Bone and Mineral Research.

## Introduction

Iron is an essential trace element used in many biological processes. The most critical of these applications is incorporation within heme for generation of hemoglobin in red blood cells and the transport of oxygen.^(^ ^1^ ^)^ Iron functions in other cellular capacities, including serving as a cofactor for numerous enzymatic reactions. Thus, iron deficiency exhibits a wide range of detrimental effects in multiple tissues and biological procedures. Iron deficiency is one of the most widespread mineral deficiencies worldwide,^(^ ^2^ ^)^ primarily caused by insufficient dietary uptake,^(^ ^3^ ^)^ inflammation‐mediated functional iron deficiency,^(^ ^4^, ^5^ ^)^ or blood loss.^(^ ^6^ ^)^ Studies in both rodents and humans show alterations in bone homeostasis during chronic iron deficiency. BMD is reduced with chronic low‐iron–diet feeding in rats.^(^ ^7^, ^8^, ^9^ ^)^ In humans, chronic iron deficiency anemia is associated with increased fracture risk, independent of other risk factors, such as hypertension or BMI.^(^ ^10^ ^)^ With aging, the incidence of iron‐deficiency anemia increases,^(11^ ^)^ and dietary iron was shown to ifluence postmenopausal women bone density parameters.^(^ ^12^, ^13^ ^)^

Bone mineralization is mediated by osteoblasts that are derived from mesenchymal stromal cells (MSCs) within the bone marrow. MSCs undergo highly coordinated differentiation programming to generate osteogenic cells capable of producing and secreting osteoid matrix for mineralization. The effects of iron deficiency using the iron chelator deferoxamine (DFO) on osteoblasts have provided controversial results. Initial studies used DFO as an hypoxia mimetic to target hypoxia‐inducible factor (HIF) stabilization, as iron is a cofactor of prolyl‐hydroxylase domain containing (PHD) proteins that signal HIF degradation.^(^ ^14^ ^)^ Activation of HIF1α in low‐oxygen tension‐culturing conditions or promotion of HIF1α accumulation through blockade of the degradation pathway, enhanced osteoblast differentiation both in vitro^(^ ^15^, ^16^, ^17^, ^18^ ^)^ and in vivo.^(^ ^19^, ^20^, ^21^ ^)^ Alternatively, iron overload is associated with impaired osteoblast activity.^(^ ^22^, ^23^ ^)^ In some postmenopausal osteoporosis studies, iron chelation is found to have a beneficial effect as this prevents iron overload^(^ ^24^, ^25^ ^)^ and potentially cell death.^(^ ^26^ ^)^

MSCs are capable of differentiation toward several cell types, including the osteogenic, chondrogenic, and adipogenic lineages.^(^ ^27^, ^28^ ^)^ Aging, in both humans and mice, exhibits an accumulation of bone marrow adipocytes,^29^, ^30^ ^)^ which is negatively associated with bone strength.^(^ ^31^ ^)^ Anemia frequently occurs during aging and in chronic diseases, yet the mechanism of disrupted bone homeostasis during iron deficiency remains unclear. In this study, we use a mouse progenitor cell line and primary human MSCs (hMSCs) to evaluate osteoblast and adipocyte differentiation in the presence of DFO to mimic chronic iron deficiency. We found that osteogenic differentiation is significantly disrupted with chronic DFO treatment despite upregulation of HIF1α and is not caused by increased cell death. Interestingly, similar DFO concentrations in adipogenic conditions failed to impede adipocyte differentiation. Therefore, osteoblasts maintain a higher iron requirement than adipocytes for proper differentiation and mineralization.

## Materials and Methods

### Mouse progenitor cells

MPC2 cells^(^ ^32^ ^)^ were grown in α modified essential medium (Hyclone) supplemented with 10% FBS (Lot nos. K19151 and B18021; Atlanta Biologicals), 1% L‐glutamine (Hyclone), and 1% penicillin/streptomycin (Hyclone) and maintained at the proliferative temperature (33°C) with 5% CO_2_. To induce differentiation, MPC2 cells were seeded at 1.5 × 10^5^ cells/mL in growth media and grown to 80% confluency. For osteogenic differentiation, growth medium was supplemented with 50μg/mL L‐ascorbic acid (Fisher Scientific) and 4mM β‐glycerophosphate (Fisher Scientific), after which cells were placed at 37°C. Adipogenic differentiation was induced with growth media supplemented with 0.1μM dexamethasone (Sigma), 5μg/mL insulin (Sigma), and 50 μM indomethacin (Sigma) and similarly transferred to 37°C. Deferoxamine mesylate (Sigma) was dissolved in water, then filter sterilized and added to the media at the indicated concentrations at the initiation of differentiation (water used as vehicle control). Media was changed every 2 to 3 days and contained all of the supplementations described above; cells were harvested at the indicated time points. Experiments included different batches of thawed cells and passages 6 to 20.

### Human progenitor cells

Human MSCs from two healthy donors (Lonza; 28‐ and 31‐year‐old men) were grown in Mesencult (Lonza) media according to the manufacturer's protocol at 37°C with 5% CO_2_ using cell passages 3 to 7 from different batches of thawed cells. To induce osteoblast differentiation, cells were seeded at 1.5 × 10^5^ cells/mL and grown to confluency, where the growth medium was replaced with osteogenic media: DMEM supplemented with 10% FBS, 1% penicillin/streptomycin, 50μM ascorbic acid, 10mM β‐glycerophosphate, and 10nM dexamethasone. For adipogenic differentiation, growth media was replaced with adipogenic media: DMEM supplemented with 10% FBS, 1% penicillin/streptomycin, 0.1μM dexamethasone, 0.5mM 3‐isobutyl‐1‐methylxanthine (IBMX; Sigma), 0.2mM indomethacin, and 10μg/mL insulin. DFO was added to the media at the indicated concentrations at the initiation of differentiation. Media were changed every 2 to 3 days and cells were harvested at the indicated time points.

### Iron supplementation

MPC2 cells and hMSCs were seeded in 12‐well plates and allowed to come to 80% confluency in appropriate standard growth media and conditions. The media for each cell type was then changed to undergo osteogenic differentiation as described above. A subset of wells was additionally supplemented with 5μM DFO. Holo‐transferrin (Sigma), the iron‐bound form of transferrin, was added to the control and DFO treated wells at two concentrations: 0.1 and 1 mg/mL. Apo‐transferrin, with no iron bound, was added to normalize transferrin protein levels so that the final total transferrin concentration remained consistent at 1.2 mg/mL across the treatments. Vehicle control was pure water. Media was changed every 2 to 3 days and RNA isolation and staining took place at the indicated time points.

### Osteoblast staining

Between 21 and 28 days of osteoblast differentiation, media was removed and washed with PBS. Evaluation of mineralization occurred with Alizarin Red (Sigma). Briefly, cells were fixed in cold 70% ethanol and stained with 2% Alizarin Red. After imaging, Alizarin Red stain was eluted with 100mM cetylpyridinium chloride and measured in a spectrophotometer at 405 nm to determine terminal mineralization. To measure functional alkaline phosphatase activity, replicate wells in a 12‐well plate were lysed (Cell Signaling) at the indicated time points, and the supernatant assayed with an alkaline phosphatase kit (Pointe Scientific) according to the manufacturer's protocol. Measurements at one‐min intervals for a total of five measurements were collected on the Epoch one‐plate reader (BioTek) and normalized to total protein levels quantified with the Bradford Coomassie assay (Pierce).

### Adipocyte staining

At 7, 14, and 21 days after adipogenic differentiation, media were removed from the plates and cells washed in PBS. The cells were then stained with 70% Oil Red O (Sigma) in propylene glycol (Fisher Scientific). Wells were rinsed until any unbound stain no longer remained and were allowed to dry. Images were collected on a Leica inverted scope using the 4× objective. Oil Red O staining then was eluted from triplicate wells in isopropanol (Fisher Scientific) and read on the Epoch One spectrophotometer plate reader at 490 nm for semiquantitative analysis of Oil Red O content.

### Cell death assay

MPC2 cells and hMSCs were plated on sterile glass chamber slides (Fisher Scientific) at 1.5 × 10^5^ cells/mL. Once the cells reached 70% confluency the medium was changed to the appropriate corresponding osteogenic media and increasing concentrations of DFO. At the indicated time points, the media was removed from the cells and the wells were washed with PBS. The slides were fixed in 10% buffered formalin and underwent fluorescent terminal deoxynucleotidyl transferase dUTP nick end labeling (TUNEL; Sigma) according to the manufacturer's instructions. DNaseI (Thomas Scientific) treatment was used as a positive control for staining as suggested by the manufacturer (Supplementary information Fig. S1). Coverslips were mounted with ProLong Gold containing 4,6‐diamidino‐2‐phenylindole (DAPI; Life Technologies) and four images per chamber were captured using a Leica fluorescent microscope. ImageJ software (NIH; https://imagej.nih.gov/ij/) was used to quantify DAPI‐positive and TUNEL‐positive cells. Each condition was conducted in two different chambers and the experiment was performed at least three times.

### Western blot

Protein was isolated from each cell type at the indicated time points from 6‐well plates in 1× cell lysis buffer (Cell Signaling) containing the protease inhibitor AEBSF (Sigma). Lysate concentrations were quantified using the Bradford Coomassie colormetric assay (Pierce), and supernatants were run on SDS‐page gels (Bio‐Rad) and transferred to polyvinylidene fluoride membranes (Bio‐Rad). Blots were probed using primary antibodies for HIF1α (1:500; Novus Biologicals) and normalized to horseradish peroxidase tagged β‐actin (1:15,000; Sigma).

### RNA analysis

RNA was isolated directly from the cells in culture using the PureLink RNA extraction kit (Invitrogen) according to the manufacturer's instructions. A total of 50‐ng RNA was then analyzed in the TaqMAn OneStep RNA to Ct kit (Life Technologies). We used TaqMan primer/probe mix (ABI) for the following genes for mouse/human: *Runx2/RUNX2*, *SP7*, *Bglap/BGLAP*, *Col1a1/COL1A1*, *Dmp1/DMP1*, *Pparɣ/PPARɣ*, *Adipoq/ADIPOQ*, and *Lpl/LPL*, which were run on a Quant Studio 3 Real‐Time PCR instrument (Life Technologies). MPC2 genes were normalized to either hypoxanthine guanine phosphoribosyl transferase (*Hprt*) or VIC‐labeled primer limited *Actb*; hMSCs were normalized to VIC‐labeled primer limited *ACTB* and calculated using the 2^‐Δ/ΔCt^ calculation method using day 1 controls as baseline. Primer/probe information is available upon request.

### Statistical analysis

Data are presented as individual data points from two to three replicates along with mean ± SD. One‐way or matched two‐way ANOVA was used when Gaussian distribution was normal for statistical analysis between doses/time. If significant values were obtained, Tukey's post hoc multiple comparisons were performed. If the Gaussian distribution failed with the D'Agostino‐Pearson test, a nonparametric one‐way ANOVA Kruskal‐Wallis test was conducted. Threshold cutoff for a significant *p* value was designated at *p* ≤ 0.05 (GraphPad Prism).

## Results

### Osteoblast differentiation requires physiologically normal iron

**Fig. 1:**
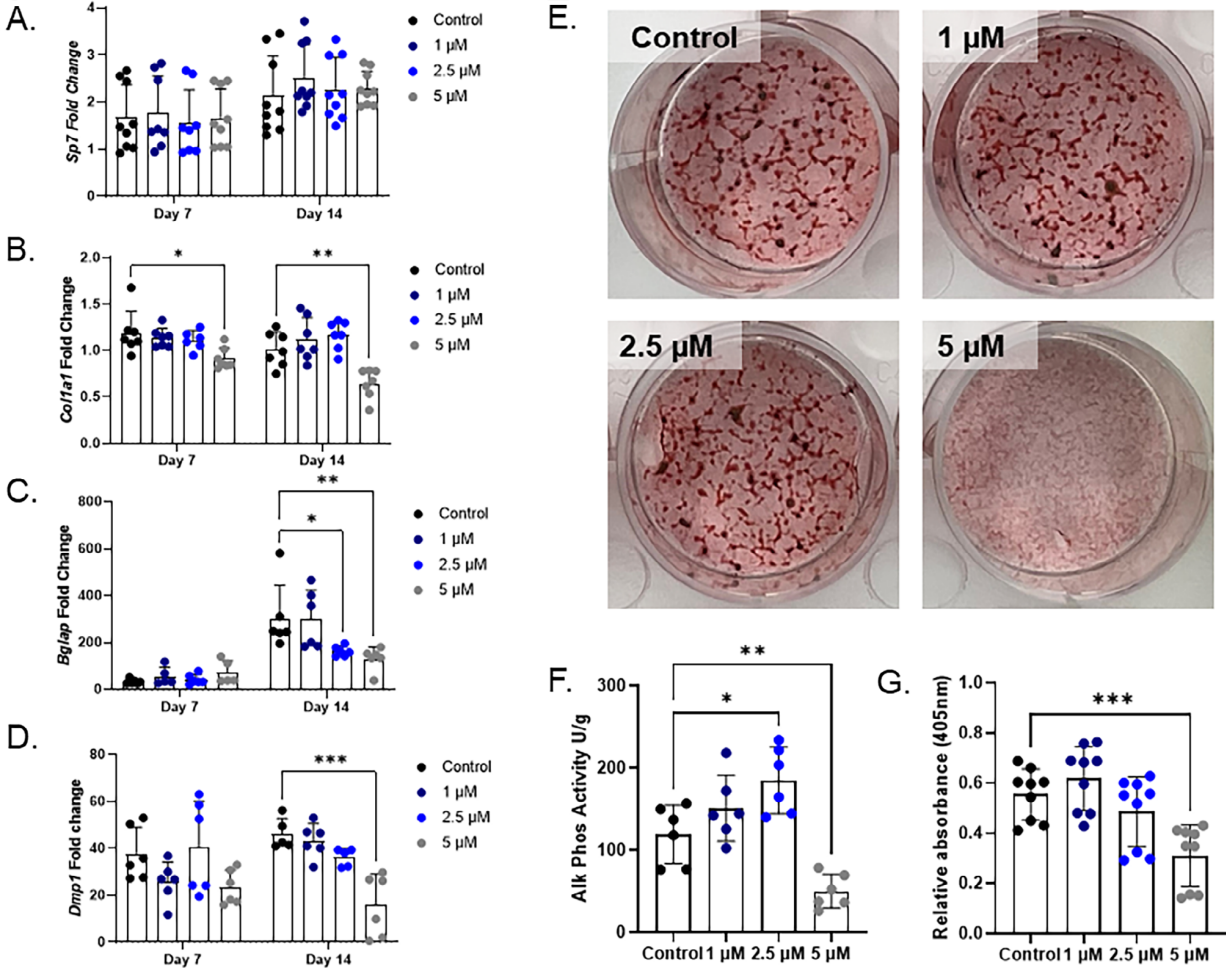
MPC2 osteoblast differentiation inhibition with deferoxamine (DFO). (*A*) *Sp7*, (*B*) *Col1a1*, (*C*) *Bglap*, and (*D*) *Dmp1* mRNA measured from undifferentiated cells or those differentiated in osteogenic media with 1, 2.5 or 5μM DFO for 7 and 14 days. (*E*) Alizarin Red staining on MPC2 cells after 21 days of osteogenic differentiated cells (*p < 0.05, **p < 0.02, and ***p < 0.001 as determined by matched two‐way analysis of variance [ANOVA]). (*F*) Alkaline phosphatase activity in osteogenic differentiated MPC2 cells at day 14. (*G*) Alizarin Red semiquantification after terminal osteoblast differentiation at day 21 (*p < 0.05, **p < 0.01, and ***p < 0.001 as determined by one‐way ANOVA).

Under osteogenic conditions, MPC2 cells exhibited expression of various osteoblast genes including osterix (Sp7), type 1 collagen (Col1a1), osteocalcin (*Bglap*), and dentin matrix protein 1 (*Dmp1*; Fig. 1*A*–*D*). The iron chelator deferoxamine (DFO) was introduced at increasing concentrations (1–5μM) at the onset of differentiation to mimic chronic iron deficiency. After 7 days of differentiation with 5μM DFO, a modest yet significant reduction in *Col1a1* mRNA was observed. After 14 days of differentiation, DFO significantly reduced *Bglap* mRNA levels in a dose‐dependent manner. Both *Col1a1* and *Dmp1* showed a significant reduction in the presence of 5μM DFO, whereas *Sp7* mRNA levels remained stable across all conditions (Fig. 1*A*–*D*). These gene‐expression changes correlated with a reduction in alkaline phosphatase activity and Alizarin Red‐stained mineralized nodules in the 5μM DFO‐treated cells versus control (Fig. 1*E‐G*).

**Fig. 2:**
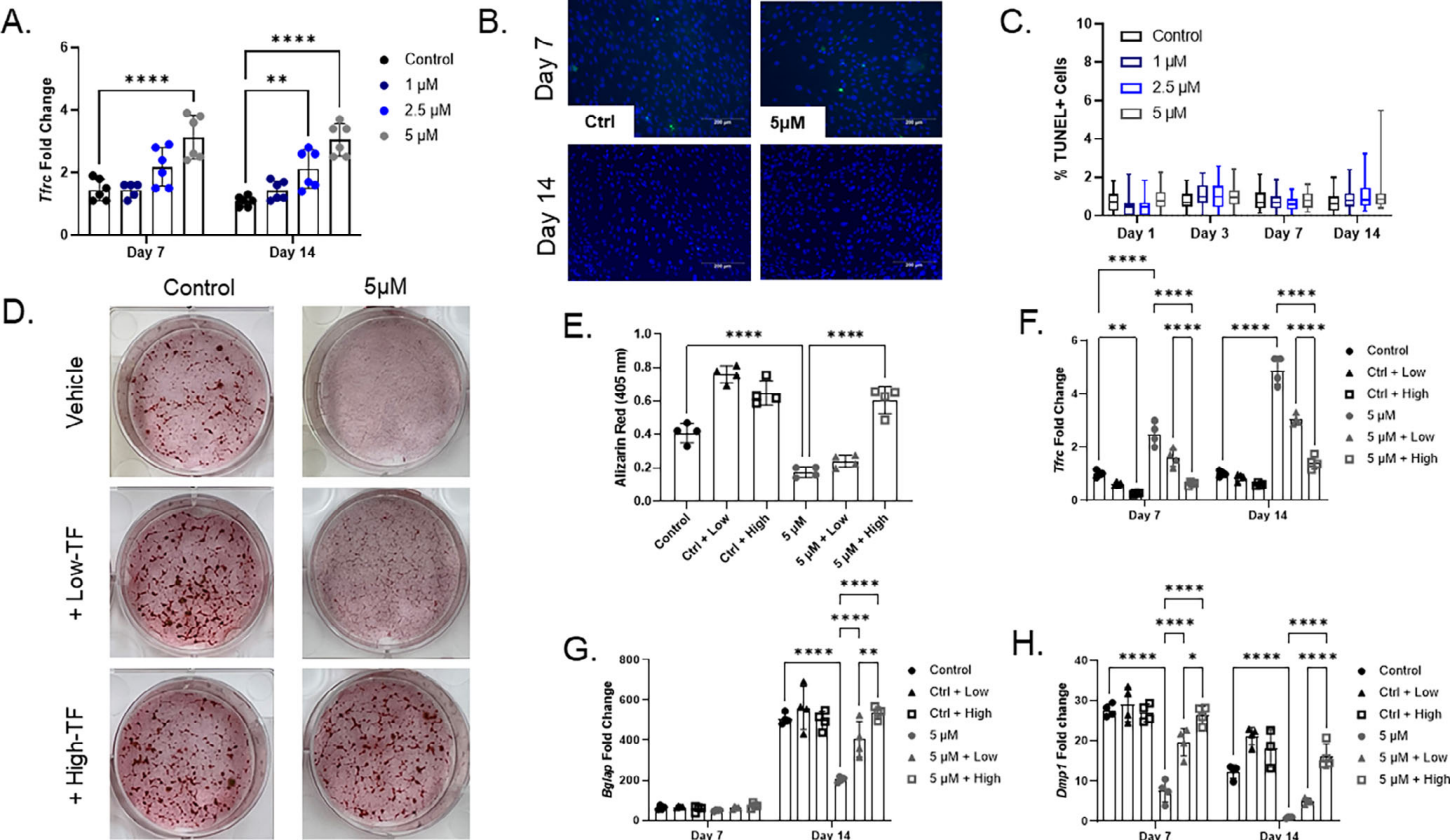
MPC2 cells treated with deferoxamine (DFO) retain iron‐adaptive responses and cell viability, as well as exhibit normal differentiation with physiological iron. (*A*) *Tfrc* mRNA measured from MPC2 cells undergoing osteogenic differentiation for 7 and 14 days with increasing DFO concentrations (**p < 0.01 and ****p < 0.0001). (*B*) Representative 10× microscopic images for MPC2 cells stained for TUNEL (green) and 4,6‐diamidino‐2‐phenylindole (blue) after 7 and 14 days of osteogenic differentiation (10×; scale bar = 200μM). (*C*) Quantitation of % dead cells after 1, 3, 7, and 14 days of osteogenic differentiation with all doses of DFO (Kruskal‐Wallis test performed at each time point). (*D*) MPC2 cells in osteoblast differentiation was supplemented with combinations of 5μM DFO with low concentration or high concentration of iron bound holo‐transferrin and stained for Alizarin Red after 21 days (*E*), which were then quantified (*p* < 0.05 based on one‐way analysis of variance [ANOVA]). (*F*) *Tfrc*, (*G*) *Bglap*, and (*H*) *Dmp1* mRNA expression levels were measured after 7 and 14 days in these conditions (*p < 0.05, **p < 0.01, and ****p < 0.0001 based on matched two‐way ANOVA).

Low iron and hypoxic states are known to increase transcription of transferrin receptor (*Tfrc*) mRNA.^(^ ^33^ ^)^ Compared with control cells, *Tfrc* mRNA showed a DFO‐dependent induction at both day 7 and day 14, suggesting that cells were attempting to adapt to the reduced iron conditions (Fig. 2*A*). Substantial iron deficiency is also known to induce apoptosis.^(^ ^34^ ^)^ To determine if blunted osteoblast differentiation was a result of cell death, MPC2 cells were cultured on glass slides and underwent TUNEL staining to quantify the level of apoptosis induced by DFO (Fig. 2*B*). After 24 hours of adding osteogenic differentiation media with or without DFO, there was no increase in TUNEL‐positive apoptotic cells in the DFO‐treated wells. At later time points, a modest increase in TUNEL‐positive cells was observed for the MPC2 osteogenic cells treated with 5μM DFO; however, it did not reach statistical significance (Fig. 2*C*). Finally, DFO is capable of chelating other metal ions; iron repletion with iron‐bound transferrin (holo‐transferrin) was used to confirm the role of iron as well as the level necessary for normal osteoblast maturation. Two concentrations of holo‐transferrin, normalized with apo‐transferrin, were supplemented in the osteogenic media with or without 5μM DFO to simulate anemic (low‐TF) or physiologically normal (high‐TF) iron concentration delivery to the cells. As observed in Fig. 1, 5μM DFO in the media for 21 days inhibited Alizarin Red staining. Importantly, only high‐TF in conjunction with the 5μM DFO restored normal mineralization (Fig. 2*D*,*E*). DFO‐treated cells showed a robust induction of *Tfrc*, which was reversed with high‐TF treatment in 5μM DFO‐treated cells at both the day 7 and day 14 time points (Fig 2*F*). Examination of osteoblast gene‐expression levels showed the 5μM DFO‐mediated reduced levels of *Bglap* and *Dmp1* were elevated in a holo‐transferrin dose‐dependent response (Fig. 2*G*,*H*).

**Fig. 3:**
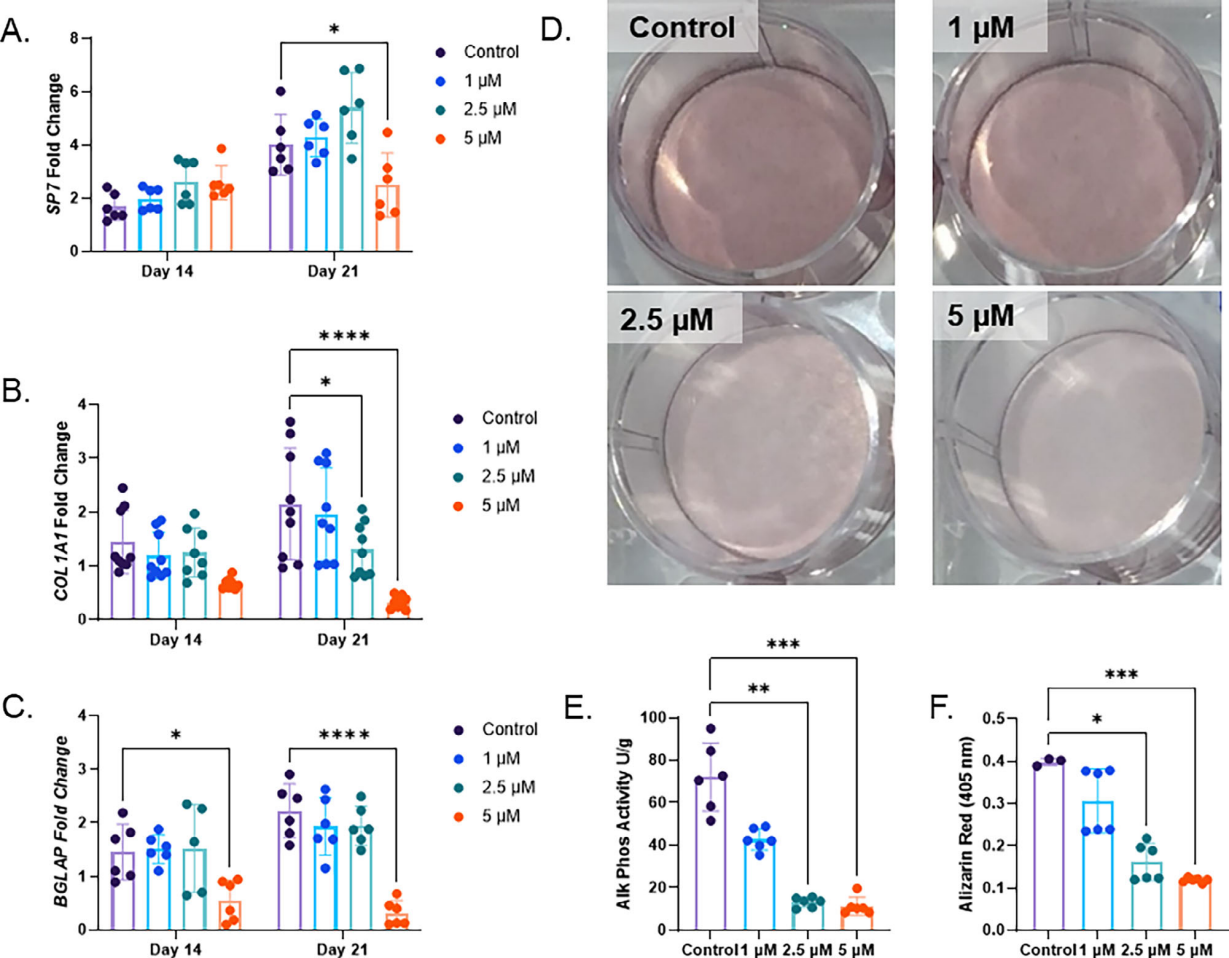
Human mesenchymal stromal cells treated with chronic deferoxamine (DFO) show suppressed osteoblast differentiation. (*A*) *SP7* (osterix), (*B*) *COL1A1*, and (*C*) *BGLAP* mRNA were measured at day 14 and day 21 after osteogenic differentiation with increasing concentrations of DFO (*p < 0.05 and ****p < 0.0001 based on matched two‐way ANOVA). (*D*) Alizarin Red staining, (*E*) alkaline phosphatase activity, and (*F*) Alizarin Red semiquantification conducted on day 21 of osteoblast differentiation with DFO (*p < 0.05, **p < 0.01, and ***p < 0.001 from a Kruskal‐Wallis test).

To investigate whether iron‐deficient inhibition of osteoblastogenesis could be caused by the species or immortalization of the MPC2 cells, primary human MSCs were differentiated toward osteoblasts with the same increasing concentrations of DFO. Similar to MPC2 cells, hMSCs exhibited expression of the osteoblast genes *SP7*, *COL1A1*, and *BGLAP* after 14 and 21 days of osteogenic differentiation (Fig. 3*A*–*C*). Increasing concentrations of DFO showed a modest decrease in *SP7* primarily observed at day 21 with 5μM DFO compared with the control cells. In contrast, *COL1A1* mRNA showed a more DFO dose‐dependent suppression of expression levels at 21 days after differentiation initiation (Fig. 3*B*). *BGLAP* mRNA levels were significantly reduced with 5μM DFO treatment for both day 14 and 21 time points (Fig. 3*C*). Similar to MPC2 cells, hMSCs showed reduced Alizarin Red staining for mineralization after 21 days, with increasing DFO concentrations in the osteogenic media (Fig. 3*D*). Both alkaline phosphate activity and Alizarin Red staining elution showed a DFO‐dependent reduction for osteoblast activity and mineralization (Fig. 3*E*,*F*).

**Fig. 4:**
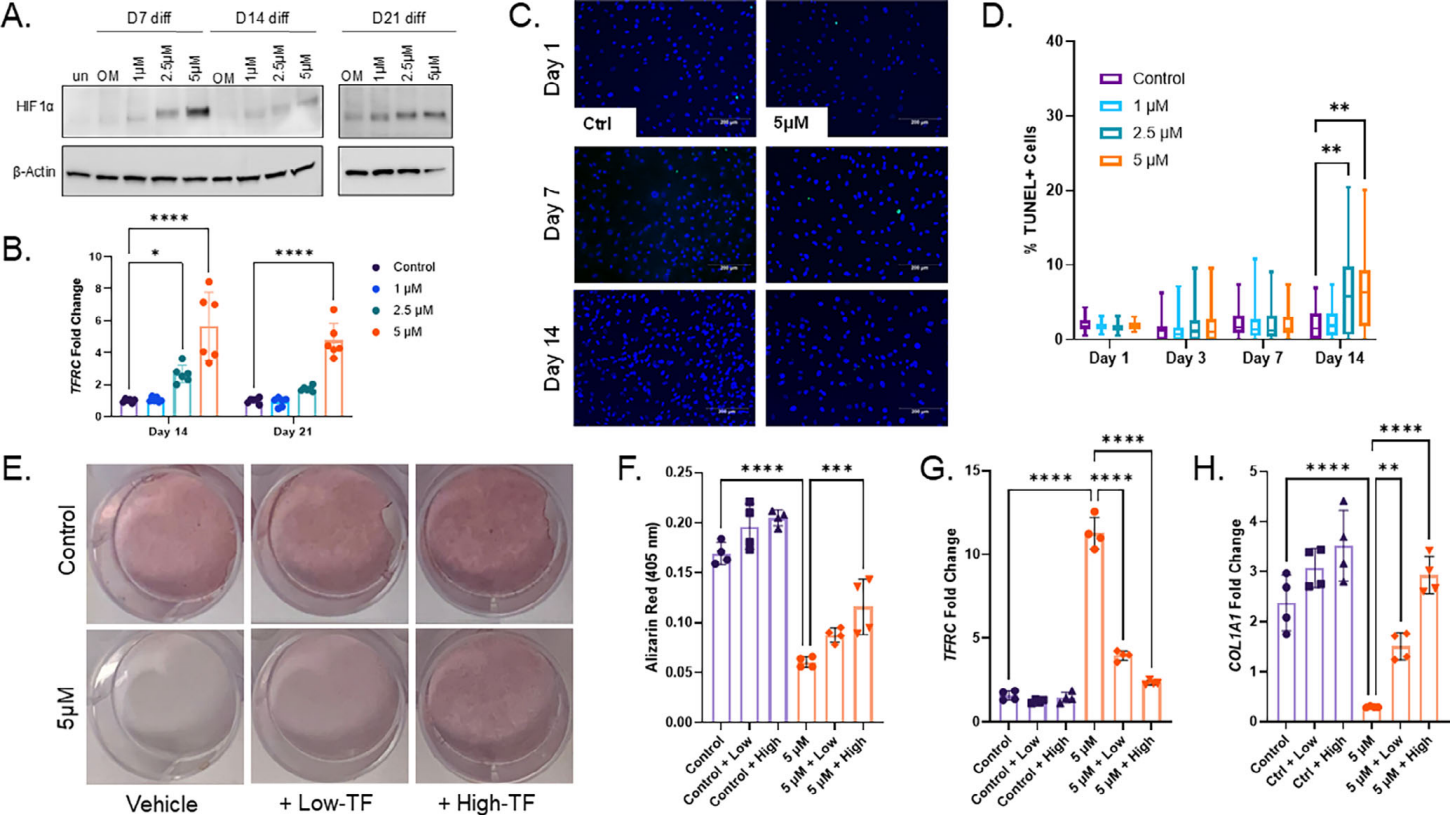
Human mesenchymal stromal cells (hMSCs) maintain iron‐adaptive responses and cell viability during chronic iron deficiency with partial rescue using iron supplementation. (*A*) HIf1α Western blot from protein isolated 7, 14, and 21 days after initiation of osteoblast differentiation, along with increasing concentrations of deferoxamine (DFO). (*B*) *TFRC* mRNA measured at 14 and 21 days of osteoblast differentiation with increasing concentrations of DFO (*p < 0.05 and ****p < 0.0001 from matched two‐way analysis of variance [ANOVA]). (*C*) Representative images (10×; scale bar = 200μM) and (*D*) quantitation for % dead cells by enumerating TUNEL‐positive (green) versus total cell (4,6‐diamidino‐2‐phenylindole [DAPI]; blue) staining after osteogenic differentiated for 1, 3, 7, and 14 days (**p < 0.01 using Kruskal‐Wallis test). (*E*) hMSCs in osteogenic media were supplemented with combinations of 5μM DFO with low concentration or high concentration of iron‐bound holo‐transferrin and stained for Alizarin Red after 28 days (*F*), which were then quantified. (*G*) *TFRC‐* and *(H*) *COL1A1* mRNA‐expression levels were measured after 21 days in these conditions (**p < 0.01, ***p < 0.001, and ****p < 0.0001 based on one‐way ANOVA).

Iron‐sensing mechanisms were also examined in these cells, including HIF1α stabilization. Protein isolated at all time points showed accumulation of HIF1α in the DFO conditions and at all of the time points examined (Fig. 4*A*). Consistent with iron deficiency and hypoxia, *TFRC* mRNA increased in parallel with increasing concentrations of DFO compared with controls (Fig. 4*B*). Again, to rule out apoptosis as a mechanism for decreased differentiation, cells at 1, 3, 7, and 14 days underwent TUNEL staining (Fig. 4*C*). Early time points showed no difference in apoptotic‐positive cells at any concentration of DFO. By day 14 of osteogenic differentiation, a modest yet significant increase in dead cell percentage was calculated for cells treated with 2.5 and 5μM DFO (Fig. 4*D*). To evaluate the level of iron needed for proper osteoblast differentiation, 5μM DFO‐treated cells were additionally supplemented with low‐ or high‐holo‐transferrin for the direct delivery of iron. After 28 days in these conditions, Alizarin Red staining showed significantly improved mineralization with 5μM DFO cells treated with high‐TF; however, these did not reach control levels (Fig. 4*E*,*F*). At the 21‐day time point, the DFO‐mediated induction of *TFRC* was reversed in a dose‐dependent manner, and the *COL1A1* mRNA levels were significantly increased also in a transferrin dose‐dependent manner (Fig. 4*G*,*H*).

### Iron deficiency exhibits less severe effects on adipogenesis

**Fig. 5:**
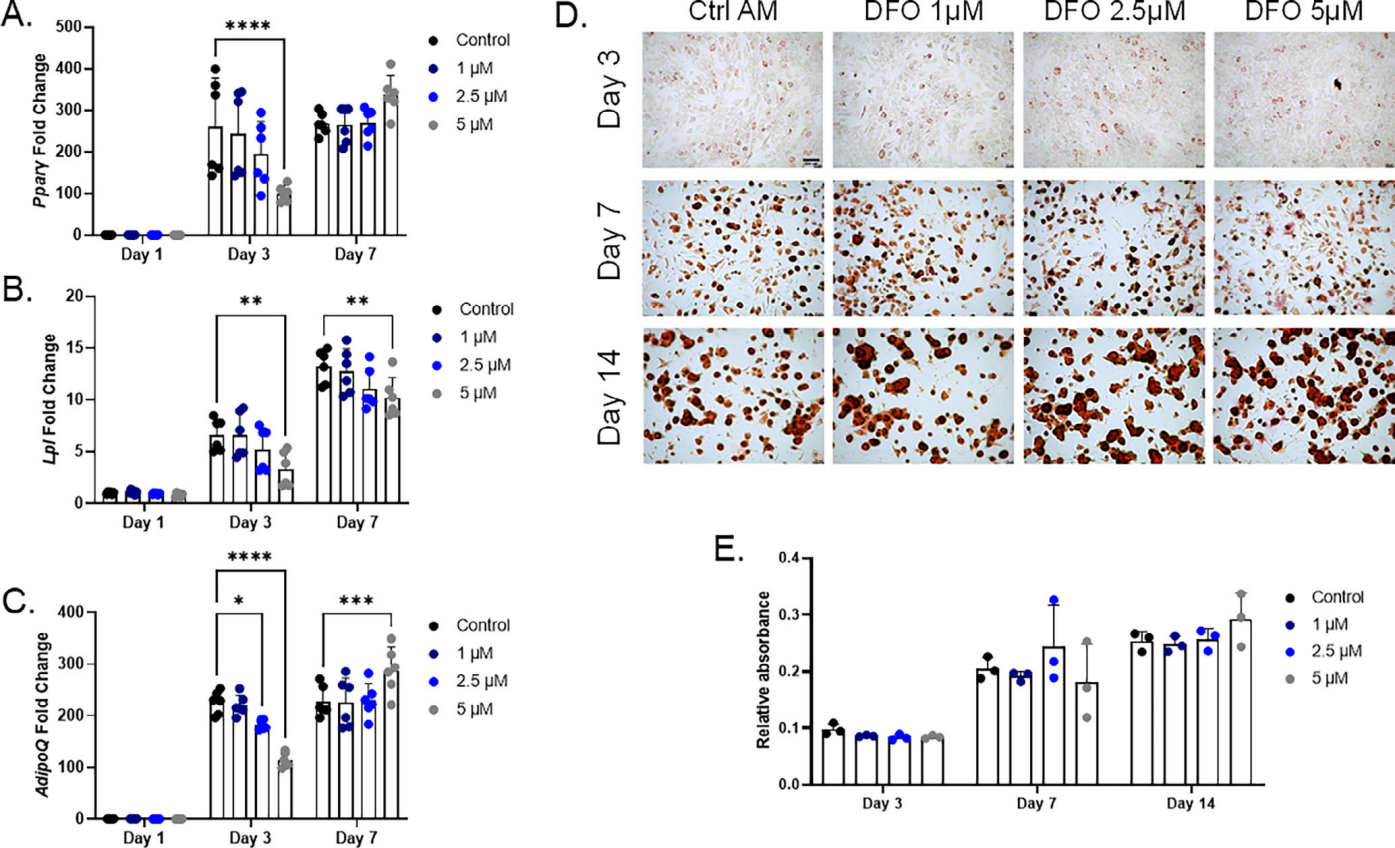
Adipocyte differentiation in MPC2 cells is not impaired with iron chelation. (*A*) *Pparγ* mRNA was analyzed at 1, 3, and 7 days after the initiation of adipogenic differentiation with increasing deferoxamine (DFO) concentrations in parallel with (*B*) *Lpl* and (*C*) *AdipoQ* mRNAs. (*D*) Representative images for Oil Red O staining and lipid accumulation after 3, 7, and 14 days of adipogenic differentiation (4×; scale bar = 100μM), which was then (*E*) eluted and semiquantified (*p < 0.05, **p < 0.01, ***p < 0.001, and ****p < 0.0001 based on matched two‐way analysis of variance). AM, adipogenic media.

As mesenchymal progenitor cells are also capable of differentiating toward adipocytes, MPC2 cells were incubated in adipogenic media (AM) with varying concentrations of DFO, similar to those used in the osteogenic differentiation conditions. PPARɣ plays a crucial role in initiating adipocyte differentiation.^(^ ^35^ ^)^ Control AM‐treated MPC2 cells showed robust induction of *Pparɣ* at both 3 and 7 days after initiation. In contrast to osteogenic differentiation, the addition of DFO had minimal effects on *Pparɣ* expression. A total of 5μM DFO‐treated cells had significantly reduced *Ppar*ɣ mRNA levels compared with control cells after 3 days of adipogenesis. However, after 7 days of adipogenic differentiation *Pparɣ* mRNA levels in the 5μM DFO conditions were similar to controls (Fig. 5*A*). As PPARɣ regulates adipogenic differentiation and gene expression, the factors lipoprotein lipase (*Lpl*) and adiponectin (*AdipoQ*) mRNA expression levels were analyzed. *Lpl* mRNA showed a significant reduction in the 5μM DFO‐treated cells at both the 3‐ and 7‐day time points compared with AM controls (Fig. 5*B*). *AdipoQ* also showed a significant reduction in the 5μM DFO‐treated cells after 3 days of differentiation. However, at day 7, *AdipoQ* mRNA levels were elevated above the controls at the same time point (Fig. 5*C*). At the cellular level, MPC2 cells showed lipid droplet accumulation, visualized through Oil Red O staining, after 3 days in adipogenic media and further increased after 7 and 14 days in the differentiation conditions (Fig. 5*D*). Semiquantitation of the eluted stain showed no difference in lipid accumulation at the various time points with DFO supplemented in the adipogenic media (Fig. 5*E*). To further evaluate a potential enhancement in adipogenesis, we reduced the levels of adipogenic components to 50% of the original concentration and repeated the MPC2 adipogenic differentiation along with DFO. *Pparγ*, *Lpl*, and *AdipoQ* mRNA levels showed a dose‐dependent decrease after 3 days of differentiation, which were normalized by day 7 (Supplementary Information Fig. S2 *A*–*C*). Importantly, Oil Red O elution after 10 days of differentiation in these conditions also showed a dose‐dependent reduction exhibiting impaired lipid accumulation (Supplementary Information Fig. S2 *D*).

**Fig. 6:**
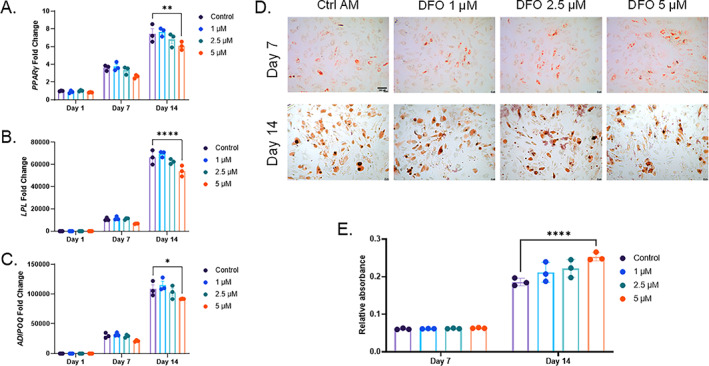
Human mesenchymal stromal‐cell adipocyte differentiation is unaffected by iron deficiency. (*A*) *PPARγ*, (*B*) *LPL*, and (*C*) *ADIPOQ* mRNAs were analyzed at 1, 7, and 14 days of adipogenic differentiation with increasing deferoxamine concentrations. (*D*) Representative images for Oil Red O staining and lipid accumulation after 7 and 14 days of adipogenic differentiation (4×; scale bar = 100μM), which was then (*E*) eluted and semiquantified (*p < 0.05, **p < 0.01, and ****p < 0.0001 based on match two‐way analysis of variance). AM, adipogenic media.

As with osteogenic differentiation, hMSCs were also incubated in AM with the same DFO concentrations. Similar to MPC2 cells, gene expression of *PPARɣ*, *LPL*, and *ADIPOQ* mRNA expression was analyzed at 1, 7, and 14 days after the initiation of adipogenic differentiation. After 14 days, the 5μM DFO‐treated cells showed significantly reduced levels of *PPARɣ*, *LPL*, and *ADIPOQ* mRNA compared with the controls, although expression levels were substantially elevated over day 1 cells (Fig. 6*A*–*C*). Oil Red O staining and semiquantitative elution of the cells at day 7 and 14 time points showed no impairment in lipid accumulation when the cells were differentiated with increasing concentrations of DFO (Fig. 6*D*,*E*).

## Discussion

Iron is a key mineral intimately involved in oxygen‐sensing mechanisms both at the cellular and systemic levels. Previous studies have found that iron levels are correlated with BMD and BMC,^(^ ^36^ ^)^ and the presence of anemia increases fracture risk independent of other known risk factors.^(^ ^10^ ^)^ This has important implications for other conditions known to induce anemia such as cancer, situations of chronic inflammation, and chronic kidney disease (CKD).^(^ ^5^, ^37^, ^38^ ^)^ Indeed, iron deficiency is highly prevalent in patients with CKD, ^37^, ^39^, ^40^, ^41^ ^)^ and these patients are found to have significantly elevated fracture risk over the general population.^(^ ^42^, ^43^, ^44^ ^)^ Both sequelae are independently associated with morbidity and mortality in CKD^(^ ^45^, ^46^, ^47^, ^48^ ^)^; however, the exact role of iron in bone homeostasis at the molecular level is unclear. The data presented here show that iron is critical for osteoblast differentiation and mineralization function. Addition of the iron chelator deferoxamine throughout the differentiation of progenitor cells toward osteoblasts mimicked chronic iron deficiency. Differentiation under DFO treatment ultimately blunted gene expression of key downstream factors necessary for appropriate differentiation. These included *Col1a1*, *Bglap*, and *Dmp1* for the mouse progenitor MPC2 cells, and *SP7*, *COL1A1*, and *BGLAP* in the hMSCs. Interestingly, osterix (*SP7*) mRNA in the hMSCs only displayed a modest reduction in expression levels and was stable in the MPC2 cells. It is possible that the mechanism of blunted osteoblast gene expression is not caused by a change in the upstream transcriptional factors: This requires further investigation. Ultimately, suppressed osteoblast gene expression led to reduced mineralization as measured by Alizarin Red staining and alkaline phosphatase activity for both cell types.

Because of the nature of the bone and bone marrow niche, osteoblasts and their progenitors reside in regions with lower oxygen tension compared with other parts of the body.^(^ ^49^ ^)^ These conditions can initiate hypoxia responses. Previous studies examining the role of hypoxia for bone homeostasis found that differentiating progenitor cells and osteoblasts in low oxygen tension conditions (3%–5% O_2_) increased osteoblast gene expression and mineralization compared with cells in normoxic conditions (21% O_2_).^(^ ^18^, ^50^ ^)^ Hypoxia‐inducible factors were deemed responsible for this effect and were modeled in vivo. Indeed, deletion of HIF1α within the osteoblast lineage decreased bone.^(^ ^51^ ^)^ Alternatively, the deletion of PHDs or von Hippel‐Lindau proteins that drive HIF degradation within the osteoblast lineage increased bone volume and density likely because of the constitutive activation of HIF transcription factors.^(^ ^52^, ^53^, ^54^ ^)^ Iron deficiency is also known to enhance HIF1α accumulation as iron, in addition to oxygen, is a critical cofactor for PHD activity.^(^ ^55^ ^)^ HIF1α protein was stabilized in the hMSCs in a DFO dose‐dependent manner during osteoblast differentiation. HIF1α stabilization was also evident after several weeks of differentiation; thus, the mechanisms to adapt were not dampened in chronic iron‐deficient conditions. Despite activation of HIF1α, however, the cells were unable to undergo osteoblastic differentiation. These data suggest that iron affects pathways in osteoblast maturation independent of HIF1α.

Known downstream targets of hypoxia and iron‐deficiency responses include transferrin receptor, which is induced in an effort to increase iron uptake. Indeed, transferrin receptor expression was upregulated in a DFO dose‐dependent manner within both of the cell types in osteogenic media. This is similar to the study by Messer et al.,^(56^ ^)^ whereby isolated fetal rat calvarial osteoblasts showed transferrin receptor induction with DFO treatment during osteogenic differentiation. To further determine whether iron is necessary for osteoblast maturation and function, we replenished the DFO‐containing media with iron in the form of holo‐transferrin. In vitro, when osteoblasts are treated with free iron in the form of ferric ammonium citrate, this has shown to induce cell death as free iron can be toxic to cells.^(^ ^57^ ^)^ Indeed, iron‐overload disorders studied in mice, including hereditary hemochromatosis, have been shown to have reduced bone compared with WT mice.^(^ ^58^ ^)^ In this way, holo‐transferrin directly delivers iron through the transferrin‐receptor–mediated pathway reducing the accumulation of free iron. Cells, differentiated toward osteoblasts but not exposed to DFO, showed little effect of holo‐transferring either at the low or high concentrations showing the iron‐bound delivery did not result in toxicity of the cells. Iron delivery in the control conditions modestly reduced *Tfrc* mRNA expression to decrease iron uptake and ultimately and modestly elevated Alizarin Red staining of these cells. When either cell type was differentiated with 5μM DFO, holo‐transferrin restored the *Tfrc/TRFC* mRNA and osteoblast gene expression in a dose‐dependent manner. In MPC2 cells, the high‐TF level of iron completely rescued mineralization, whereas hMSCs mineralization rebounded more modestly.

Using primary hMSCs and mouse progenitor cells allowed for the assessment of iron on alternative differentiation pathways. During aging, there is an inverse relationship between bone marrow adipocyte accumulation and bone strength within the vertebrae.^(^ ^29^ ^)^ As 5μM DFO in the osteogenic culture media was able to significantly suppress osteoblast differentiation, we sought to determine whether there would be a reciprocal increase in adipogenesis. Concentrations of DFO, found to inhibit osteoblastogenesis, exhibited a very minimal effect in hMSC or MPC2 cells cultured with adipocyte differentiation media. Indeed, iron‐deficiency conditions neither hindered nor enhanced adipogenesis. The expression of key adipocyte genes showed modest reductions at the highest DFO concentration. At the cellular level, DFO did not impair the ability of either cell type to accumulate lipid throughout the adipogenic differentiation protocol. In fact, the 5μM DFO‐treated hMSCs were observed to have moderately increased Oil Red O levels compared with controls. To further evaluate the potential for iron deficiency to enhance adipogenic differentiation we reduced the adipogenic components in the media. We found under these conditions, iron deficiency inhibited Oil Red O accumulation. This is partially in line with the study by Mareno‐Navarrete et al.^(^ ^59^ ^)^; the authors found that DFO dramatically inhibited *PPARɣ*, *ADIPOQ* mRNA expression in human preadipocytes. However, the concentrations of DFO were 4‐ to 20‐fold higher than those used in our study. Additionally, it is unclear how the iron contained within the media, provided by the supplemented FBS, may have differed between the two studies. These data suggest that when iron deficiency outweighs adipogenic signals, adipogenesis is impaired.

Early in osteogenic differentiation, osteoblasts have high energetic demands, requiring robust ATP production.^(^ ^60^, ^61^, ^62^ ^)^ Mitochondria play an essential role in this process; importantly, iron is used as a cofactor for many enzymes within these organelles.^(^ ^63^ ^)^ Thus, alterations in iron could interfere with the mitochondrial biology and cellular energy metabolism during osteoblast differentiation, which has been implicated in other tissues.^(^ ^64^ ^)^ The requirement for appropriate iron concentrations for mitochondrial function could explain our results, whereby only physiologically normal iron concentrations supplemented media rescued mineralization when in the presence of DFO. However, whether iron‐mediated changes in energy metabolism directly link to alterations in osteoblast gene expression remains to be determined. In contrast, adipocytes have a distinct metabolic profile to that of osteoblasts,^(^ ^65^ ^)^ which may possibly render them less sensitive to the effects of 5μM DFO‐mediated iron depletion on mitochondrial dynamics. Interestingly, in mice subjected to iron deficiency, bones display reduction in bone density yet have not exhibited marrow fat accumulation compared with controls.^(^ ^66^ ^)^ Therefore, based on our in vitro culture data, this suggests that iron deficiency is not sufficient to directly promote adipocyte differentiation over the osteoblast pathway. In the context of dietary iron restriction, it is possible that either the total number of progenitor cells is reduced or there is a lack of factors within the bone marrow microenvironment to stimulate adipogenic differentiation.

In summary, these data suggest that osteoblasts maintain a higher iron requirement for differentiation and function compared with adipocytes, and that additional external stimuli are needed to promote adipocyte differentiation during frank iron deficiency. Additionally, mouse and human progenitor cells display similar phenotypes when differentiated under chronic iron‐deficiency states. By understanding the molecular alterations induced by iron deficiency, this could have important implications for helping to mitigate fracture risk in situations of chronic low iron.

## Conflict of Interest

The authors have nothing to disclose.

### PEER REVIEW

The peer review history for this article is available at https://publons.com/publon/10.1002/jbm4.10529.

## Supporting information

**Figure S1** Positive control for TUNEL staining. Each cell type underwent treatment with DNaseI for 10 minutes to induce DNA breaks prior to staining. Representative 10X (scale bar = 100 μM) images for MPC2 (left) and hMSC (right) are included.

**Figure S2** Iron deficiency inhibits adipogenic lipid accumulation with reduced adipogenic components. MPc2 cells were supplemented with 50% lower concentrations of adipogenic components in combination with DFO plus vehicle. (*A*) *Pparγ*, (*B*) *Lpl* and (*C*) *AdipoQ* mRNAs were analyzed at 1, 3, and 7 days of adipogenic differentiation (*P < 0.05 and **p P< 0.01, based on match two‐way ANOVA). (*D*) Oil red O stain at day 10 of differentiation was eluted and semi‐quantified (*P < 0.05, ***P < 0.001 and ****P < 0.0001 based on one‐way ANOVA).
