# Supplementary figures and images for: Differential Iron Requirements for Osteoblast and Adipocyte Differentiation
Source: JBMR Plus. 2021 Jul 26;5(9):e10529. doi: 10.1002/jbm4.10529 (PMC8441506; doi:10.1002/jbm4.10529)

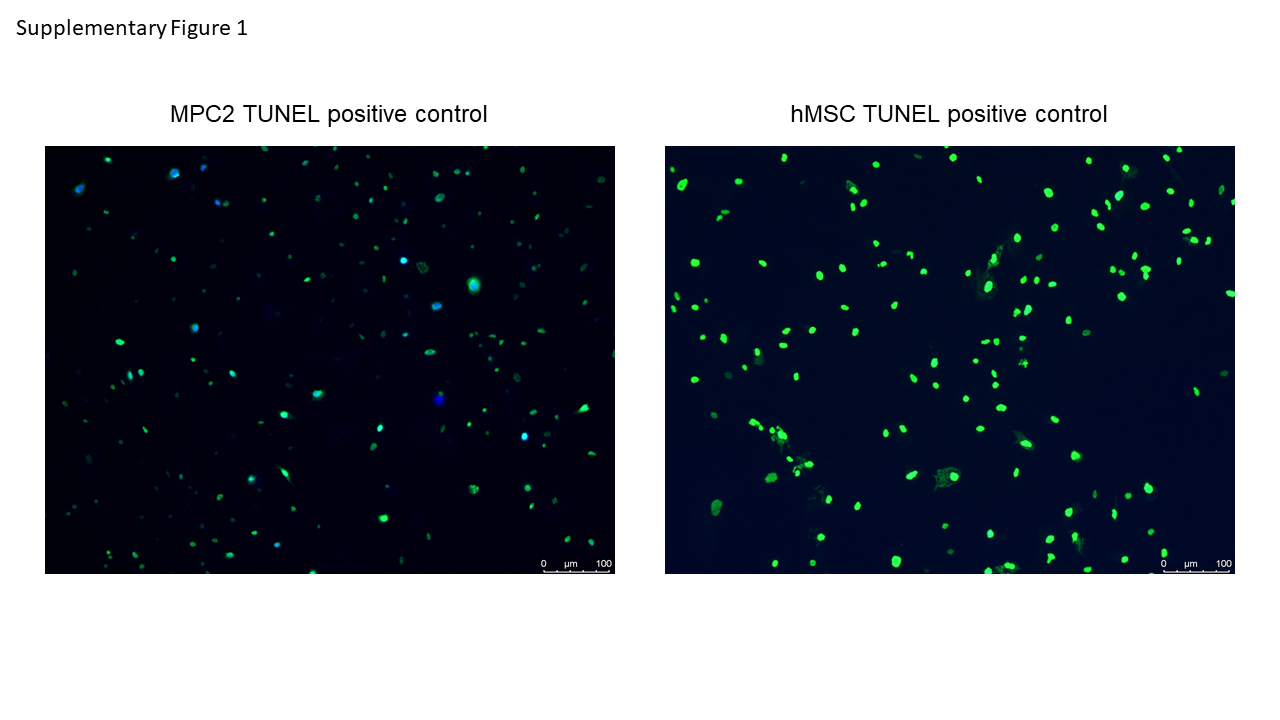

Supplement: Supplementary file 1 — Figure S1 Positive control for TUNEL staining. Each cell type underwent treatment with DNaseI for 10 minutes to induce DNA breaks prior to staining. Representative 10X (scale bar = 100 μM) images for MPC2 (left) and hMSC (right) are included. [file JBM4-5-e10529-s002.TIF]

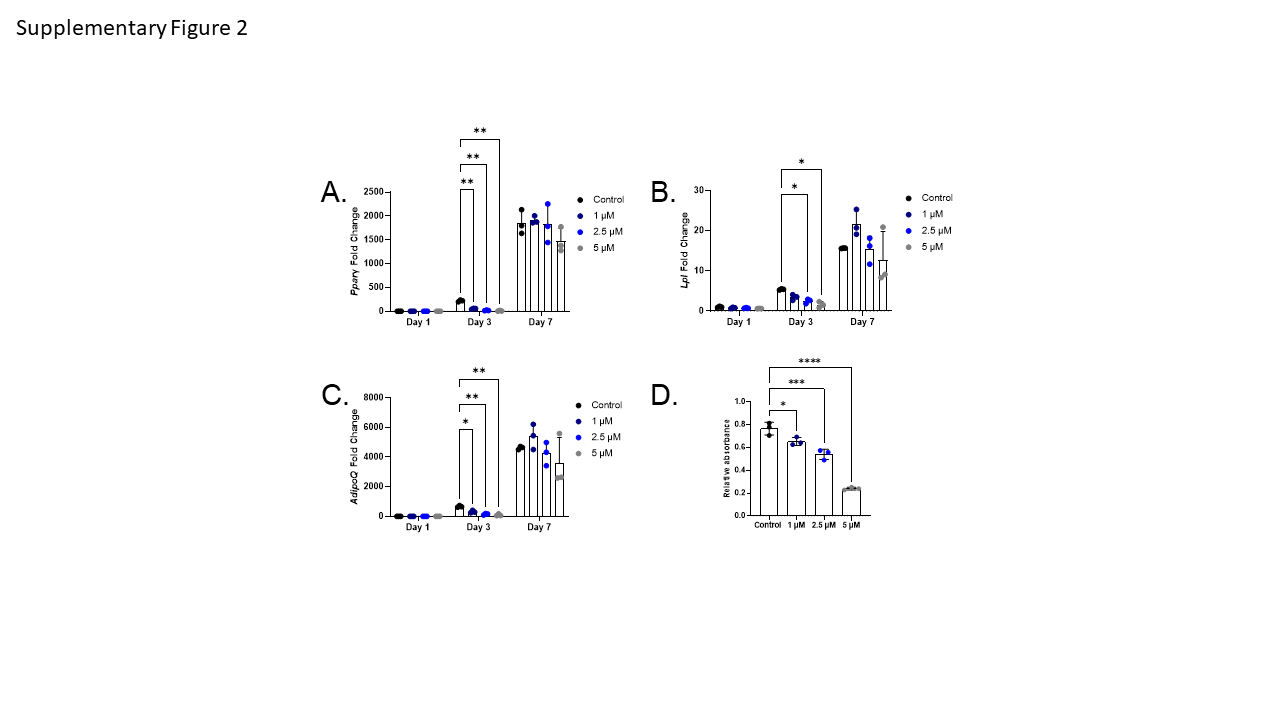

Supplement: Supplementary file 2 — Figure S2 Iron deficiency inhibits adipogenic lipid accumulation with reduced adipogenic components. MPc2 cells were supplemented with 50% lower concentrations of adipogenic components in combination with DFO plus vehicle. (A) Pparγ, (B) Lpl and (C) AdipoQ mRNAs were analyzed at 1, 3, and 7 days of adipogenic differentiation (*P < 0.05 and **p P< 0.01, based on match two‐way ANOVA). (D) Oil red O stain at day 10 of differentiation was eluted and semi‐quantified (*P < 0.05, ***P < 0.001 and ****P < 0.0001 based on one‐way ANOVA). [file JBM4-5-e10529-s001.TIF]
